# A *Mangifera indica* L. Extract Could Be Used to Treat Neuropathic Pain and Implication of Mangiferin

**DOI:** 10.3390/molecules15129035

**Published:** 2010-12-09

**Authors:** Bárbara B. Garrido-Suárez, Gabino Garrido, Rene Delgado, Fe Bosch, María del C. Rabí

**Affiliations:** 1 Laboratorio de Farmacología Molecular del Centro de Investigación y Desarrollo de Medicamentos, Ave. 26 No. 1605, Nuevo Vedado, Havana, Cuba; 2 Clínica del Dolor Hospital Docente Clínico Quirúrgico 10 de Octubre, Calzada de 10 de Octubre, 130 entre Alejandro Ramírez y Agua Dulce, Havana, Cuba; 3 Departamento de Química y Farmacia, Facultad de Ciencias, Universidad Católica del Norte, Antofagasta, Chile

**Keywords:** neuropathic pain, neuroimmune mechanisms, Vimang, *Mangifera indica* L*.*, mangiferin

## Abstract

It has been accepted that neuroinflammation, oxidative stress and glial activation are involved in the central sensitization underlying neuropathic pain. Vimang is an aqueous extract of *Mangifera indica* L. traditionally used in Cuba for its analgesic, anti-inflammatory, antioxidant and immunomodulatory properties. Several formulations are available, and also for mangiferin, its major component. Preclinical studies demonstrated that these products prevented tumor necrosis factor α -induced IκB degradation and the binding of nuclear factor κB to DNA, which induces the transcription of genes implicated in the expression of some mediators and enzymes involved in inflammation, pain, oxidative stress and synaptic plasticity. In this paper we propose its potential utility in the neuropathic pain treatment. This hypothesis is supported in the cumulus of preclinical and clinical evidence around the extract and mangiferin, its major component, and speculates about the possible mechanism of action according to recent advances in the physiopathology of neuropathic pain.

## Abbreviations

sGC: soluble guanylyl cyclise
PLA_2_: phospholipase A_2_
AA: arachidonic acid
COX: cyclooxygenase
PKA: protein kinase A
P2X4: purinergic P2 receptor
CX3CR1: chemokine fractalkine receptor
NMDA: N-methyl-D-aspartate receptor
AMPA: α-amino-3-hydroxy-5-methylisoxazole-4-propionic
mGluRs: metabotropic glutamate receptors
NK1: tachykinin NK1 receptor
TNF-R: TNF receptor

## 1. Introduction

It was estimated that 37.6 million individuals suffered from neuropathic pain across the seven major markets in 2005 and it is forecasted that its prevalence will increase to 39.1 million individuals by 2011 [1]. Unfortunately neuropathic pain is often resistant to available drug therapies directed at receptors and channels on neurons, including opioids, topical analgesic, anti-epileptics, *N*-methyl-D-aspartate (NMDA)-antagonists and tricyclic antidepressants, none of which achieve clinical significance greater than 50% [2]. To address this problem it is necessary an alternate and complementary approach to thinking about and treating neuropathic pain involving neuroimmune and glial activation, in particular for its active participation in central sensitization, a pivotal mechanism in persistent pain [3]. Recently it has been suggested that the tetrapartite synapse, which includes an astrocyte, a microglial cell, and pre-and post-synaptic neuronal terminals, is a critical functional unit, that contributes to its generation and maintenance [4]. In line with this evidence, the use of specific cytokine inhibitors, anti-inflammatory cytokines, transcription factor inhibitors or glial modulators has shown anti-hyperalgesic effect in neuropathic and inflammatory pain animal models [3,4]. On the other hand, there is some evidence that reactive oxygen species (ROS) and cytokine production play a role in neuropathic pain [2]. ROS production and antioxidant mechanisms of compensatory enhancement were observed in the spinal cord after peripheral nerve injury [5]. Many studies in animal models and humans showed the anti-hyperalgesic and anti-allodynic effects of ROS scavenging agents [5,6]. Also, recent studies have established that the analgesic action of Vitamin E occurs through desensitization of neurons and inactivation of NMDA receptors in neuropathic rats [7]. It has been recognized that elevated levels of ROS play a key role in the phosphorylation of NMDA receptor subunit 1 (pNR1) through to protein-kinases activation in the spinal dorsal horn (SDH) [7,8]. Tumor necrosis factor *alpha* (TNFα) has been implicated in both, mechanical and thermal hyperalgesia as well [3,9,10,11]. This cytokine contributes to the release of nitric oxide (NO), ROS, eicosanoids and glutamate and induces C fibres long term potentiation (LTP) on SDH in nerve injury rats. This effect was reverted for transcription nuclear factor *kappa* B (NF-κB) inhibitors [12,13]. Moreover, TNFα and interleukin 1 *beta* (IL-1β) may be inducing damage by inhibiting glial cells ability to remove glutamate from the synaptic cleft [3,4,14]. Subsequently, neuroimmune activation and oxidative stress proposes new targets for therapeutic intervention in neuropathic pain [4,14]. 

Vimang is the brand name of a standard aqueous stem bark extract of selected varieties of mango (*Mangifera indica* L.; MSBE), which contains a definite mixture of components including poly-phenols, triterpenes, phytosterols, fatty acids and microelements [15]. This pharmaceutical active ingredient is used to produce Vimang tablets (300 mg) and a 1.2 % cream which have been protected by patent [16] and are registered as a phytodrug, food supplement or cosmetic by the Cuban health regulatory agencies. Previous experiments on that extract have shown that it has antioxidant [17], anti-inflammatory, analgesic [18,19] and immunomodulatory [20,21] properties. This extract prevents TNFα-induced IκB degradation and the binding of NF-κB to the DNA, which induces the transcription of genes implicated in the expression of some mediators and enzymes involved in inflammation, pain, oxidative stress and synaptic plasticity [22,23,24]. Highly significant are the liver- and brain-protecting effects of MSBE on rats and gerbils, respectively, in ischemia-induced models [25,26]. Mangiferin, its major component (about 15-20% from the extract), shows neuroprotective effects in glutamate induced neuronal and glial injury model and antioxidant activity related to its iron-chelating properties in addition to scavenging activity of free radicals has been reported [27,28,29]. In addition it is able to limit microglial activation in terms of attenuation of prostaglandins E_2 _(PGE_2_) production, free radicals formation and reduction of cyclooxygenase-2 (COX-2) synthesis induced by lipopolysaccharide [30]. This evidence suggest the potentiality of this compound to modulate some of the molecular targets implicated in peripheral and central neuropathic pain mechanisms, especially central sensitization [31]. Therefore, it is necessary to implement neuropathic pain models and link the behaviors with biochemical determinations, biological molecular assays and immunohystochemical studies to establish the molecular action of these products on specific targets in several signaling pathways involved in neuropathic pain [32]. In accordance with preclinical results clinical trials in complex regional pain syndrome (CRPS), zoster-associated pain (ZAP), diabetic polyneuropathy, and other painful peripheral neuropathies could be performed. Some preliminary results in clinical case reports and case series in CRPS and ZAP have been published, but more preclinical and clinical evidence are yet necessary to demonstrate this hypothesis [33,34]. This paper focuses on unifying the cumulus of evidences around the MSBE and mangiferin, hypothesizing about their mechanism of actions on neuropathic pain according to recent advances in its knowledge.

## 2. The Antinociceptive Action of MSBE in Formalin-induced Licking and Acetic acid-induced Writhing Models in Mice

The ED_50_ of MSBE for the late phase (15-30 min) of nociception in the 1% formalin model was 8.4 mg/kg b.w., but the early phase (0-5 min) was not affected. In acetic acid-induced writhing in mice, a somato-visceral pain model, the ED_50_ was 54.5 mg/kg b.w. [18]. Both licking and writhing nociceptive behavior are mediated by NMDA receptor activity, known to be associated with nitric oxide (NO) synthesis [31]. 

The exclusive inhibition of phase II of formalin 1% test for the MSBE is an element that suggests its capacity to modulate a pathological and persistent painful status more than acute nociceptive pain. The formalin test is widely used as a model of acute inflammatory pain, as formalin activates peripheral sensory nerves and produces pain behaviors that involve ongoing peripheral activity (phase I) and peripheral and central sensitization (phase II) underlying in hyperalgesia [35]. It has also been established that ROS play a role in this model; in peripheral sites, its action may be more important mediating the acute phase; whereas the spinal site is the key mediating phase II [36]. Mechanisms of phase II are dose-related over range 0.5-5% and the participation of glutamate-NMDA-nitric oxide synthase (NOS)-NO in the spinal neuronal circuits changes has been demonstrated [35,37]. Moreover, the Ca^2+^ influx induced for NMDA activity triggers enzymatic cascades, which finally also evoke the release of prostaglandins [38,39]. Subsequently, the selective effect that shows COX and NOS inhibitors and NMDA antagonist in this phase as well as in neuropathic pain model is known, where central sensitization is involved [40,41]. On the other hand, an increase in expression of mRNA and protein has been reported for the transient receptor potential vanilloid type 1 receptor (TRPV1) in dorsal root ganglia (DRG) of uninjured afferents in neuropathic pain models, as well as the lack of changes in thermal or mechanical hypersensitivity in TRPV1 knockout mice after partial sciatic nerve ligation (PSL) [42,43]. In addition, it has been demonstrated recently that this receptor regulates glutamatergic synaptic inputs to the spinothalamic tract neurons of the spinal cord deep dorsal horn [44]. Consequently, several clinical trials have demonstrated the efficacy of its agonists such as capsaicin cream (0.025-0.075%) and NGX-4010, a high concentration (8%) capsaicin patch, as topical agents for the treatment of post-herpetic neuralgia [45,46], which can lead to excitation and desensitization of nociceptive afferents. Subsequently, antagonists have also been evaluated as potential analgesics in clinical trials [47]. The formalin test at low concentration is mediated by activity of capsaicin-sensitive neurogenic components [40]. MSBE showed antihyperalgesic and anti-edematogenic effects in phase II of formalin test at 1% [18] and Vimang formulations decreased the area of dynamic allodynia in CRPS and ZAP patients [33,34]. This is an outcome frequently utilized in preclinical and clinical studies as an indicator of central sensitization maintained by peripheral inputs [48]. This extract, inhibited leukotriene B_4_ (LTB_4_) and PGE_2_ synthesis *in vitro* and showed an inhibitory effect of topical arachidonic acid- or PMA-induced edemas, in *in vivo* models sensitive to test inhibitors of lipoxygenase and cyclooxygenase, respectively [19,49,50]. The lipoxygenase products have been identified as TRPV1 activators [51]. Moreover, it is recognized that a novel mechanism modulating the TRP complexes function, which depend on protein phosphorylation [47]. Several agents, including phorbol esters, (like PMA), IL-1β and proteases, which are modulated for MSBE, have been shown to enhance the response of the TRPV1 via activation of protein kinase C (PKC). In addition, also is accepted a facilitator effect on TRPV1 receptor of prostaglandins and glutamate depending on cAMP-protein kinase A (PKA), which also are modulated by MSBE [51]. There could be a possible peripheral antihyperalgesic mechanism of action of MSBE.

## 3. The Effects of MSBE and Mangiferin in Different *in Vitro* and *in Vivo* Models of Inflammation

MSBE shows anti-inflammatory activity in different inflammatory *in vivo* and *in vitro* models in addition to its antioxidant effects [19,22,49,50]. A decisive contribution to this action could be the modulator effect of the extract and mangiferin on pro-inflammatory cytokines, such TNFα, IL-1β, granulocyte/macrophage colony stimulating factor (GM-CSF) [23] and adhesion molecules (ICAM-1) [51], chemotaxis and proliferation of T cells [22]. In addition, MSBE inhibits inducible nitric oxide synthase (iNOS) and COX-2 expression, secretory phospholipase A_2_ (PLA_2)_ activity and downstream, the activation of arachidonic acid derived-species biosynthesis, PGE_2_ and LTB_4_ [19,49,50]. These effects could be the result of actions exerted by the different polyphenolic species included into the extract, principally mangiferin [24]. But, the selective prevention of the expression of inflammation-related genes by means of NF-κB translocation inhibition is the key in its primary anti-inflammatory effect [23,24].

## 4. The Possible Role of MSBE and Mangiferin as Modulators of the Central Sensitization Mechanism

It is possible to speculate that MSBE could modulate some molecular targets implicated in central sensitization. Perhaps, its ability to inhibit TNFα synthesis, make this molecule attractive for this hypothesis. There is some evidence that involves this pro-inflammatory cytokine in this process. TNFα level increases dramatically after nerve injury and would thus cross the axonal membrane. The resulting cation channel would allow sodium to leak into the fibre and this would probably create sufficient depolarization to trigger an impulse [32]. Correspondingly, its intra-sciatic application in rats produces ectopic discharges in C and Aβ fibres; besides, anti-TNFα neutralizing antibodies or TNFα synthesis inhibitors relieve neuropathic pain in chronic constriction injury (CCI) and PSL models [3,10]. In addition, it is reported that in mice both mechanical and thermal hyperalgesia following brachial plexus avulsion (BPA), were abolished in TNFα p55 receptor knockout mice [11]. Clinical trials have begun to show that inhibition of TNFα relieves some types of neuropathic pain [53]. TNFα promote the synthesis of substance P (SP), adenosine, calcitonin gene-related peptide (CGRP) and nerve growth factor (NGF) as well as, modulate genes implicated in expression of others cytokines (IL-1, IL-2, IL-6, IL-8, and IFNγ) and induce their synthesis [14]. IL-1β may contribute to excitotoxic state, indirectly through the release of NO, ROS, eicosanoids and glutamate [3]. Additionally, it has been recognized that ROS, TNFα and IL-1 may induce damage for inhibiting glial cells' ability to remove glutamate from the synaptic cleft [4]. Then, the actions of MSBE and mangiferin on expression of TNFα and IL-1β previously demonstrated [23,33,49], could explain the antiallodynic effect and decrease of burning spontaneous pain frequency in pilot studies [33,34]. In correspondence with this findings on microglial cell line, it was demonstrated the suppression of TNFα and NO for MSBE and mangiferin, as well as more recently, their protective effect in two *in vitro* models of neuronal and glial glutamate-induced injury [49,54]. However, the molecular mechanism of mangiferin induced-neuroprotection mediated by NF-κB regulation has been reported [27,28]. This polyphenol also inhibits COX-2 expression and PGE_2 _production in activated rat microglial cells [30]. Accordingly MSBE and its principal component could be potentially glial modulators. 

In addition, TNFα induces LTP of C fibres in SDH in rats with nerve injury and NF-κB inhibitors blocked this effect [12]. NF-κB is constitutively expressed in the brain; however, its expression increases after spinal cord injury and can be activated by many different stimuli, such as pro-inflammatory cytokines, NGF, PKC and glutamate [12,31]. Subsequently, NF-κB regulates the expression of several proteins involved in nociception and neural plasticity such as pro-inflammatory cytokines, chemokines, iNOS, COX-2, and pro-dynorphin [55]. Dynorphin could modulate the immune function of microglia and astrocyte, as well as interact with NMDA receptor in neurons and produces allodynia [56]. NF-κB is a key in the regulation of the inflammatory process in reactive glial cells, its transgenic inhibition attenuates pain and inflammation after peripheral nerve injury [57]. Therefore, the primary inhibitory effect of MSBE on TNF-induced activation of NF-κB could suggest the antiallodynic effect of MSBE [3,23]. 

In addition, ROS are also involved in central sensitization, and it has been assumed that elevated levels of ROS play an important role in the phosphorylation of NMDA receptor subunit 1 (pNR1) through to PKA and PKC activation in the spinal cord [7,8]. MSBE exhibits a potent antioxidant effect depending of its free radical scavenging and iron-complexing abilities [28]. The synergistic effect of the extract could explain the advantage of MSBE over classical antioxidants (vitamins C and E and beta-carotene) in liver and brain [17]. 

## 5. Conclusions

**Figure 1:**
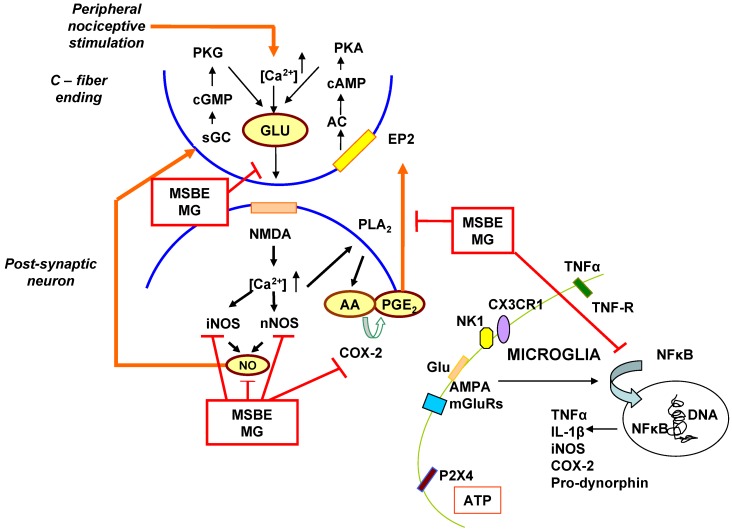
Schematic diagram illustrating the hypothetical molecular mechanism of *Mangifera indica* L aqueous stem bark extract (MSBE) and its isolated glucosylxanthone mangiferin (MG)in spinal cord nociceptive processing. Modified diagram according to Vetter *et al*. [38].

Based on the summarised evidence about *Mangifera indica* L extract (like Vimang formulations) and its major component mangiferin, we hypothesize that these formulations could be useful in treating and preventing neuropathic pain. The possible mechanism of action may be modulating several molecular targets implicated in central sensitization (Figure 1). Other peripheral, central and specific targets of diseases could also be regulated in special glial activation and immune system. Further studies must be focused on the neuroimmune mechanisms and clinical evidences in neuropathic pain syndromes treatment.

Pre-synaptic neuron stimulation leads to an enhanced release of excitatory neurotransmitters such as glutamate (Glu). Glutamate binding to post-synaptic NMDA receptors induces Ca^2+^-influx thereby initiating a cascade, which finally evokes the release of nitric oxide (NO) and prostaglandin E_2_ (PGE_2_). NO produced by neuronal NOS (nNOS) and inducible nitric oxide synthase (iNOS) penetrates membranes and may travel to neighbouring neurons, glial cells and pre-synaptic nerve terminals where it may activate second messenger systems. This results in increased level of cGMP with the subsequent activation of cGMP-dependent kinases, which in turn may lead to further glutamate release. PGE_2 _may activate adenylyl cyclase (AC) via binding to EP2 receptors resulting in the formation of cAMP, that it may activate cAMP-dependent PKA, which may induce further glutamate release [38]. Activation of the NF-κB signaling pathway by tumor necrosis factor *alpha* (TNFα) is inhibited by MSBE and MG. TNFα stimulates the degradation of NF-κB cytoplasmatic inhibitor (IκB). After this, NF-κB is translocated to the cell nucleus to induce the mRNA synthesis of some pro-inflammatory mediators such as TNFα, interleukin 1 *beta* (IL-1β), granulocyte/macrophage colony stimulating factor (GM-CSF), iNOS, inducible isoform of cyclooxygenase (COX-2) and the adhesion molecule ICAM-1 that are inhibited by MSBE and MG. The extract and its xanthone also inhibit PLA_2 _activity, PGE_2 _and leukotriene B_4 _production. MSBE and MG may inhibit glutamate release in pre-synaptic neuron and regulate microglia activity by means of NF-κB signalling pathway inhibition. 
